# Serum from humans on long-term calorie restriction enhances stress resistance in cell culture

**DOI:** 10.18632/aging.100584

**Published:** 2013-07-27

**Authors:** Daniela Omodei, Danilo Licastro, Francesco Salvatore, Seth D. Crosby, Luigi Fontana

**Affiliations:** ^1^ Division of Geriatrics and Nutritional Science, Washington University School of Medicine, St. Louis, MO 63130, USA; ^2^ CEINGE-Biotecnologie Avanzate scarl, Napoli, Italy; ^3^ CBM Scrl - Genomics, Area Science Park, Basovizza, Trieste, Italy; ^4^ Fondazione SDN-IRCCS, Napoli, Italy; ^5^ Department of Genetics, Washington University School of Medicine, St. Louis, MO 63130, USA; ^6^ Department of Medicine, Salerno University School of Medicine, Salerno; Italy

**Keywords:** calorie restriction, oxidative stress, aging, stress resistance

## Abstract

Calorie restriction (CR) without malnutrition is the most robust intervention to slow aging and extend healthy lifespan in experimental model organisms. Several metabolic and molecular adaptations have been hypothesized to play a role in mediating the anti-aging effects of CR, including enhanced stress resistance, reduced oxidative stress and several neuroendocrine modifications. However, little is known about the independent effect of circulating factors in modulating key molecular pathways. In this study, we used sera collected from individuals practicing long-term CR and from age- and sex-matched individuals on a typical US diet to culture human primary fibroblasts and assess the effects on gene expression and stress resistance. We show that treatment of cultured cells with CR sera caused increased expression of stress-response genes and enhanced tolerance to oxidants. Cells cultured in serum from CR individuals showed a 30% increase in resistance to H_2_O_2_ damage. Consistently, SOD2 and GPX1 mRNA, two key endogenous antioxidant enzymes, were increased by 2 and 2.5 folds respectively in cells cultured with CR sera. These cellular and molecular adaptations mirror some of the key effects of CR in animals, and further suggest that circulating factors contribute to the CR-mediated protection against oxidative stress and stress-response in humans as well.

## INTRODUCTION

Calorie restriction (CR) without malnutrition is the most potent and reproducible intervention for slowing aging and protecting against cancer in mammals [1,2]. Some of the beneficial effects of CR are mediated by down-regulation of inflammatory pathways and up-regulation of genes promoting stress resistance, DNA and cellular repair, and antioxidant enzymatic activity [3]. It has been hypothesized that changes in circulating factors may contribute, at least in part, to some of these beneficial effects of CR in rodents [4]. However, nothing is known on the molecular and cellular adaptations induced by circulating factors in men and women practicing long-term severe CR.

The importance of circulating factors in modulating health and longevity is supported by data obtained by heterochronic parabiosis experiments in mice [5]. Exposure of old mice to circulating factors present in young serum increased neurogenesis and regeneration after muscle injury [6,7]. It is well known that senescent stromal cells secrete a number of factors that can promote cancer and aging by increasing proliferation and tumorigenesis of epithelial cells, and by inhibiting stem cell function and antioxidant capacity [8]. It is our working hypothesis that CR by changing factors present in the systemic milieu (i.e. serum) can influence cell function by modulating key signaling pathways.

To test this hypothesis we have evaluated the effects of serum collected from volunteers practicing long-term CR on global gene expression and on stress resistance in cell culture experiments. In these in vitro experiments, we compared the molecular and cellular effects on human primary fibroblasts cultured of media supplemented with human sera obtained from: 1) healthy lean and weight-stable volunteers who were consuming CR diets, with more than adequate intake of protein and vitamins, for an average of 7 years, and 2) age-, sex-matched, non-obese individuals eating Western diets (WD).

## RESULTS

### Gene expression analysis shows up-regulation of genes involved in NRF2-mediated oxidative stress response

**Figure 1:**
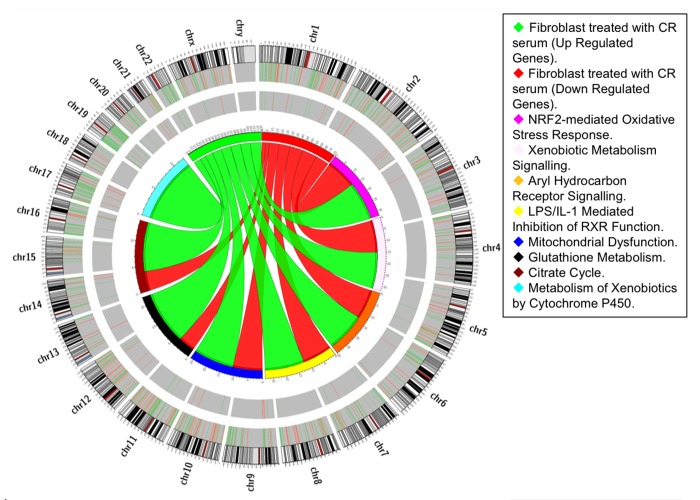
Pathway-based analysis. Circos diagram shows the relationship between CR differentially expressed genes and oxidative-related pathways. Ribbon ends represent links between genes and pathways while the width of the ribbon correlates with the number of genes involved. Segments in the outer ring indicate the total number of genes involved in the corresponding pathways while thin internal segments indicate the number of up-regulated (light green) and down-regulated (orange) genes.

**Figure 2:**
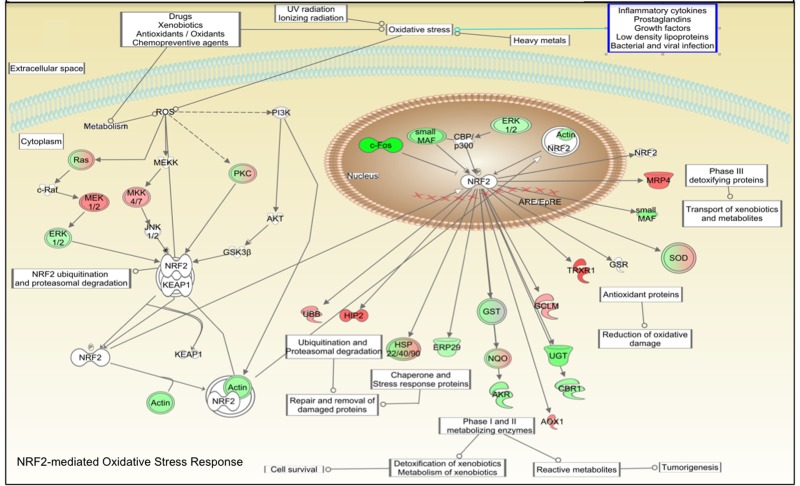
Connectivity map of NRF2-mediated oxidative stress response adapted from IPA software. Pathways analysis identified the NRF2-mediated oxidative stress response pathway with a statistically significant value (BH p-value 3.33E-02). Green colored shapes represent up-regulated genes while red ones represent down-regulated genes of fibroblast cell lines treated 48 hours with CR serum.

**Figure 3:**
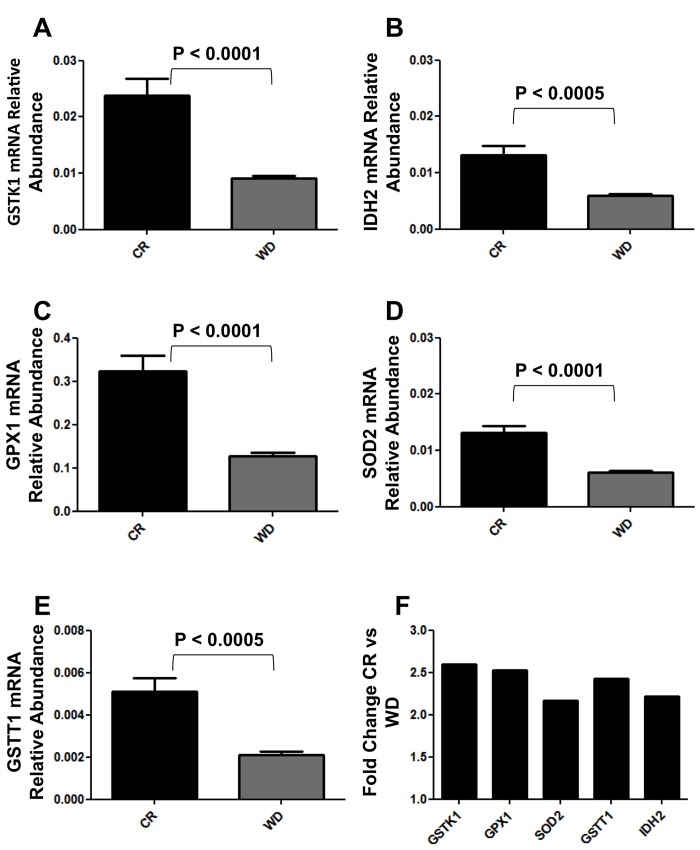
Real Time qPCR analysis of GSTK1, GSTT1, IDH2, GPX1 and SOD2 expression in BJ cells cultured 48hs with CR and WD sera. (**A**-**E**) Data are expressed as the gene of interest abundance relative to the GAPDH (Mean ± SEM); *n* = 12 CR, *n* = 13 WD. Student's t test was used to determine P-values. (**F**) Data are expressed as fold changes relative to CR cultures.

Mammalian fibroblasts have been extensively used to assess global transcriptional changes induced by circulating factors (i.e. serum) on cell function [9]. To test the hypothesis that the CR-induced changes in circulating factors can influence cell function, we compared the transcriptional profiles of human primary fibroblasts treated with CR or WD serum. Differentially expressed genes were selected using a moderate t-test approach [10] and multiple test correction [11], and these genes were used to identify transcriptionally altered pathways. Ingenuity Pathways Analysis software (IPA) showed a significant association of a subgroup of 83 genes (Supplementary Table S1) with many oxidative response-related pathways (Fig. 1). In particular, NF-E2-related factor 2 (Nrf2)-mediated oxidative stress response contained 30 genes that were dysregulated in the treated fibroblasts (BH p-value 3.33E-02), and many of them were direct expression effectors of Nrf2 activity (Fig. 2). Meta-analysis performed using a competitive gene set test [12] pinpoints 11 molecular signatures significantly down-regulated in a ‘self-contained’ gene test (Table 2 red Highlights). To further investigate the result of microarray data and confirm the association with oxidative response-related pathways, we performed quantitative real-time PCR for the following genes which were shown by microarray to be detectable and significantly dysregulated: GSTK1, GPX1, SOD2, GSTT1, and IDH2. As shown in Figure 3, relative abundance of message for these genes was significantly higher in the CR group than the control group (p<0.0005) with a fold change difference ≥ 2.2-fold.

**Table 1:** Characteristics of the study subjects

	CR group (n=12)	WD group (n=13)	P value
**Age (yrs)**	57.8±8.4	55.7±8.7	ns
**Sex (M/F)**	10/2	11/2	
**Height (m)**	1.73±0.07	1.76±0.12	ns
**Weight (Kg)**	57.1±6.6	79.8±14.0	0.0002
**BMI (kg/m^2^)**	19.1±1.2	25.7±2.3	P<0.0001
**Total body fat (%)**	9.8±4.8	25.9±5.6	P<0.0001

Values are means ± SD

**Table 2:** Gene Set Enrichment CAMERA and ROAST results

Gene Set Enrichment results.		Correlation Adjusted Mean Rank Gene				Rotation gene set testing for linear models					
Molecular Signature	NGenes	Cor	Down	Up	TwoSided	Adj.P.Value.Down	Adj.P.Value.Up	Adj.P.Value.Mixed	Active.Proportion.Down	Active.Proportion.Up	Active.Proportion.Mixed
*PROTEIN_MATURATION*	17	0.027	0.9976	0.0024	0.00	1.00	0.10	0.00	0.18	0.59	0.76
*DNA_FRAGMENTATION_DURING_APOPTOSIS*	31	−0.002	0.9951	0.0049	0.01	1.00	0.07	0.00	0.13	0.42	0.55
*AMYLOID_PRECURSOR_PROTEIN_METABOLIC_PROCESS*	24	0.005	0.9948	0.0052	0.01	1.00	0.07	0.00	0.08	0.38	0.46
*HYDROLASE_ACTIVITY_ACTING_ON_CARBON_NITROGE N_BUT_N0T_PEPT1DE_B0NDS_IN_LINEAR_AMIDES*	24	0.005	0.9925	0.0075	0.02	1.00	0.10	0.00	0.13	0.29	0.42
*MITOT1C_ CELL_ CYCLE_ CHECKPOINT*	45	0.012	0.0076	0.9924	0.02	0.06	1.00	0.01	0.31	0.09	0.40
*RAS_ GTPASE_ BINDING*	35	−0.010	0.0074	0.9926	0.01	0.04	1.00	0.01	0.34	0.14	0.49
*HOMEOS TASIS_ OF_NUMBER_ OF_ CELLS*	36	−0.009	0.0069	0.9931	0.01	0.03	1.00	0.00	0.31	0.08	0.39
*U12 DEPENDENT SPLICEOSOME*	10	−0.069	0.0063	0.9937	0.01	0.03	1.00	0.01	0.40	0.20	0.60
*APICAL_PLASMA_MEMBRANE*	25	−0.004	0.0056	0.9944	0.01	0.04	1.00	0.04	0.24	0.04	0.28
*Stress_response_CR*	11	0.002	0.0052	0.9948	0.01	0.03	1.00	0.00	0.55	0.09	0.64
*INSOLUBLE_FRACTION*	28	−0.003	0.0046	0.9954	0.01	0.03	1.00	0.01	0.29	0.04	0.32
*DNA_DAMAGE_RESPONSE_SIGNAL_TRANSDUCTION_BY _P53_CLASS_MEDIAT0R*	31	−0.002	0.0043	0.9957	0.01	0.03	1.00	0.01	0.32	0.10	0.42
*APICAL_PART_OF_CELL*	29	−0.004	0.0042	0.9958	0.01	0.03	1.00	0.06	0.24	0.03	0.28
*SMALL_CONJUGA TING_PROTElN_BINDING*	21	−0.015	0.0040	0.9960	0.01	0.03	1.00	0.02	0.33	0.05	0.38
*AMINOPEPTIDASE_ACTIVITY*	19	0.005	0.0015	0.9985	>0.01	0.03	1.00	0.00	0.47	0.00	0.47
*UBIQUITIN_BINDING*	14	−0.016	0.0008	0.9992	>0.01	0.03	1.00	0.01	0.50	0.07	0.57

### Incubation with human CR serum increases stress resistance to hydrogen peroxide in vitro

**Figure 4:**
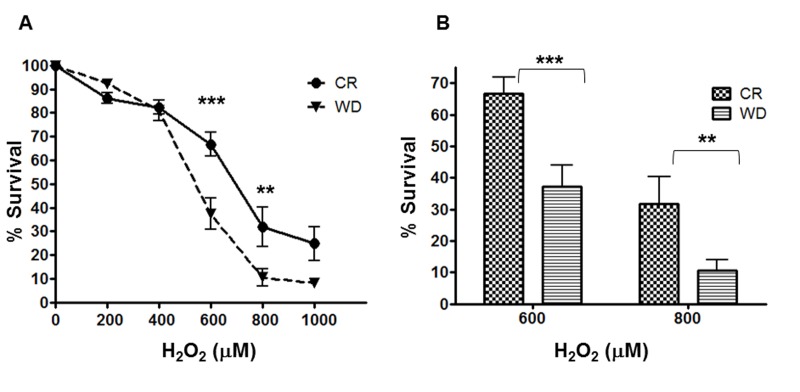
Differential Stress Resistance to hydrogen peroxide. A human fibroblast cell line (**B**J) was cultured 48-hrs with sera from CR and WD individuals. Cell viability (WST1 assay) was determined after a 24 h treatment with H_2_O_2_ (0 – 1000 M). Data are represented as means ± SEM. P- values were determined with 2way ANOVA with Bonferroni post test. ***P < 0.001; **P < 0.01

To confirm the gene expression findings with a functional test, human primary fibroblasts cultured for 48 hours with CR or WD sera were exposed to increasing concentrations of the powerful oxidizing agent, hydrogen peroxide (H_2_O_2_) for 24 hours. As shown in Figure 4, treatment with H_2_O_2_ produced a dose-dependent decline in survival, but fibroblasts cultured with CR serum were significantly protected from the cytotoxic effect of the oxidant. At 600 μM about 67% of cells pretreated with CR serum were viable, while only 37% of cells pretreated with WD serum survived (p< 0.001), and even at higher H_2_O_2_ concentrations (800 μM) 32% of the cells pretreated with CR serum survived, while only 10% of the cells exposed to WD serum were still alive (p< 0.01).

## DISCUSSION

We report here that some molecular and functional adaptations of CR, such enhanced stress resistance, can be mimicked in vitro by culturing cells with serum from individuals practicing long-term CR without malnutrition. Our findings show that the incubation of human fibroblasts with CR sera induces a transcriptional activation of several anti-oxidant genes along the Nrf2 pathway. In addition, we found that the incubation of fibroblasts with human CR serum is sufficient to increase stress resistance to hydrogen peroxide in vitro. These molecular and cellular adaptations are consistent with findings in CR rodents and monkeys, and may contribute at least in part to the protective anti-aging effects of CR [1,3].

In particular, accumulating data indicate that aging is associated with a down-regulation of stress-response genes and pathways [3]. To test the hypothesis that CR can counteract some of these age-associated detrimental effects *in vitro*, we first sought to determine if circulating factors of CR sera modify gene expression profiles of fibroblasts, which are the most abundant cell type in connective tissues and form the structural framework of tissues through their secretion of extracellular matrix components. To this end, we cultured human primary fibroblasts with sera from 12 middle-aged (57.8±8.4 yrs), weight-stable very lean (BMI=19.1±1.2 kg/m^2^) individuals who have been practicing ~30% CR with adequate nutrition (at least 100% of RDI for each nutrient), for 3-20 years, and a control group of 13 non-obese (BMI=25.7±2.3 kg/m^2^) age-matched sedentary controls eating a typical Western diet. Remarkably, we found that culturing cells with human CR sera for 48-hrs was sufficient to trigger a rapid transcriptional up-regulation of important genes and pathways that control stress resistance. In particular, we found that a highly significant number of transcripts along the Nrf2 pathway were altered by CR, and genes encoding important antioxidant enzymes, such as GSTK1, GSTT1, IDH2, GPX1 and SOD2, were up-regulated at least two fold in the CR group. Nrf2 is a key transcription factor that binds to the antioxidant response element (ARE) in response to environmental carcinogens or low levels of insulin, and increases the transcription of a variety of antioxidative and carcinogen-detoxification enzymes that boost protection against cellular and molecular damage and cancer [14-16]. It might be also possible that CR causes oxidative stress and therefore induce a NRF2-dependent response, supporting the hypothesis that oxidative damage is not a cause of aging [17-19].

In order to further investigate the role of CR sera in modifying the resistance of cells to oxidative stress, we next performed a series of experiments in which we exposed human primary fibroblasts to increasing concentrations of H_2_O_2,_ a powerful oxidizing agent. Our data indicate that incubating fibroblasts with CR sera exerts a rapid and profound protection against hydrogen peroxide-induced cytotoxicity. These findings are consistent with data from studies in mice, suggesting that these selected cellular adaptations induced by circulating factors are conserved among species [4]. Indeed, most of the metabolic and hormonal parameters examined in CR humans are similar to those reported in CR rodents [20-22].

In conclusion, our findings reported here show that enhanced stress responsiveness known to occur in animals on CR following oxidative stress can be reproduced in vitro by culturing cells using serum from CR patients. Our findings provide support for a possible neuroendocrine based mechanism of CR and offer a model for the screening and investigation of CR mimetic agents. We anticipate that this novel in vitro model will be instrumental in the elucidation of biochemical, molecular and cellular mechanisms underlying the anti-aging effects of CR in humans.

## MATERIALS AND METHODS

### Human subjects

Human serum samples were collected in the morning after an overnight fast from twelve individuals of the Calorie Restriction Society who had been practicing severe CR with adequate nutrition for an average of 7 years (range 3-20 years). Thirteen healthy, non-obese and sedentary (regular exercise < 1 h per week) individuals eating typical Western Diets (WD) were used as a sedentary comparison group; they were matched with the CR group in terms of age, sex and height. Whole blood was collected into sterile vacutainer tubes, allowed to clot for 30 minutes and then centrifuged. The serum was then stored at −80°C. The characteristics of the participants are shown in Table 1. All of the study participants were weight stable, i.e. < 2 kg weight change in the preceding 6 months. None of the participants had evidence of chronic disease (including cardiovascular, lung, gastrointestinal, type 2 diabetes and cancer). None were smokers. This study was approved by the Human Studies Committee of Washington University School of Medicine, and all participants gave informed consent before their participation.

### Cell culture

The BJ human fibroblast cell line was obtained from ATCC and used in these experiments. BJ cells were cultured in 75 cm^2^ culture flasks containing basic culture medium: 86% of E-MEM medium supplemented with 10% fetal bovine serum (Sigma-Aldrich, St. Louis, MO, USA), 1% Sodium Pyruvate, 1% non-essential amino acids, 1% GlutaMAX and 1% antibiotics (GIBCO, Carlsbad, CA, USA) until the time of treatment. Cells were cultured in a humidified incubator at 37 °C and 5% CO_2_. For the experiments, FBS was replaced with human serum from CR and WD participants. Both FBS and human serum were heat inactivated for 30 minutes at 55 °C.

### Microarray analysis

BJ cells were seeded at a concentration of 4.000 cells/cm^2^ in 25 T-25 flasks; when they reached 60% of confluence, the cells were starved for 24h without FBS. After starvation, 10% of human serum from 12 CR and 13 WD individuals was added to the cultures for 48h. Whole cells were detached from the flask with trypsin/EDTA (Sigma-Aldrich, St. Louis, MO, USA) and Total RNA was extracted using the mirVANA (Ambion, Inc, Austin, TX, USA) according to manufacturer's protocol. Integrity of total RNA was evaluated using capillary electrophoresis (Bioanalyzer 2100, Agilent technologies, Santa Clara, CA, USA) and quantified using a Nanodrop 1000 (Nanodrop, Wilmington, DE). Aliquots of RNA (400 ng) samples were amplified according to the specifications of the Illumina® TotalPrep™ RNA Amplification Kit (Ambion, Austin, TX, USA) to produce a pool of biotin-labeled RNA corresponding to the polyadenylated (mRNA) fraction. The cRNA samples were applied to the arrays of Illumina whole-genome HumanHT-12 v 3.0 (Illumina, San Diego, CA, USA) and hybridized according to the manufacturer's specification. Each array on the BeadChip targets over 25.000 annotated genes using 3-micron beads bearing covalently attached 50-base oligonucleotide probes. Each probe interrogates a single gene, and each bead type is represented with an average 15-fold redundancy on every array. The BeadChips were scanned with the Illumina's Beadarray system scanner (Illumina, San Diego, CA, USA). The hybridization images signal intensity was extracted and background subtracted using Illumina Inc. BeadStudio software version 3.3.7. The produced data were checked for the Illumina internal quality control and loaded into Bioconductor software [23,24] for statistical analysis using lumi package [25] (see below). The data discussed in this publication have been deposited in NCBI's Gene Expression Omnibus [26] and are accessible through GEO Series accession number GSE41790.(http://www.ncbi.nlm.nih.gov/geo/query/acc.cgi?token=nnonbsicqouiilw&acc=GSE41790).

### Real Time PCR

Real-time PCR was used to verify microarray data. Total RNA was DNase I treated and reverse transcribed using the High Capacity RNA-to-cDNA kit (Applied Biosystems, Carlsbad, CA, USA). The cDNA was then used to evaluate the relative expression of select genes including SOD2, GPX1, IDH2, GSTK1 and GSTT1 using specific TaqMan Gene Expression Assays (Applied Biosystems, Carlsbad, CA, USA), which are pre-designed and pre-optimized gene-specific probe sets. DNA amplification was performed using the Applied Biosystems 7500 Fast Real-Time PCR system according to the manufacturer's protocol. The relative amount was calculated from the threshold cycles with the instrument's software (SDS 2.0, Applied Biosystems, Carlsbad, CA, USA), according to the manufacturer's instructions. We used the comparative cycle threshold (Ct) method (2 - CT) to calculate relative gene expression under experimental and control conditions normalized to GAPDH. The results are expressed either as the abundance of the gene of interest relative to the house keeping gene (2 - CT), or as fold change over control values (2 - CT).

### Cell Proliferation assays in response to Hydrogen Peroxide

To determine the Differential Stress Resistance to hydrogen peroxide of serum from CR and WD individuals, human fibroblasts were seeded in 96-well plates at a concentration of 3000 cells/well in 86% D-MEM low glucose w/o phenol red supplemented with 1% Sodium Pyruvate, 1% non-essential amino acids, 1% GlutaMAX and 1% antibiotics (GIBCO, Carlsbad, CA, USA). After cells had seeded, media was removed and replaced with media containing 10% of human serum from 11 CR and 11 WD individuals was added in triplicate to the plates for 48hs. After incubation, freshly prepared hydrogen peroxide was added to the plates at a concentration of 200, 400, 600, 800, 1000 μM and cells were challenged for 24hs.

Cell viability was determined by the addition of 20 μl of the ready-to-use WST1 reagent (Roche diagnostics, Mannheim, Germany) to each well; the cells were incubated for approximately 3 hours at 37 °C and 5% CO_2_ in a humidified incubator. After this incubation period, the absorbance of the formazan dye formed was measured at a wavelength of 450 nm using a microplate spectrometer. The measured absorbance directly correlates to the number of viable cells.

### Statistical analysis

Microarray data produced were quality checked with arrayQualityMetrics [24] package under R version 2.15.1 Bioconductor software [23] and undetectable and low detection value probes were filtered out from further analysis accordingly to the lumi package standards [25]. To identify differentially expressed genes, based on a moderate t-test, the Limma package [10] was used and genes were selected based on a p-value cut-off <0.05 (after FDR adjustment) and absolute fold change >1 following the Macroarrays Quality Control Program criteria [11]. IPA software was then used (www.ingenuity.com) to detect the enrichment of biofunctions and networks in the resulting list. Core analysis was performed using the following settings: Reference set: HumanHT-12 v 4.0 [v3 is mentioned in the methods]; Relationship to include: Direct and Indirect; Includes Endogenous Chemicals; Filter Summary: Consider only molecules and/or relationships where (species = Uncategorized (e.g. chemicals) OR Human) AND (confidence = Experimentally Observed). Canonical pathways analysis has been performed using as scoring method B-H Multiple Testing Correction p-values set to a threshold value of 0.05.

Meta-analysis was performed using the CAMERA function implemented in the Bioconductor Limma package for a ‘competitive gene set test’ [12]. The resulting top Molecular signatures have been further analyzed using a ‘focused’ gene set test ROAST [27]. All the list of genes organized as Signature and present in The Molecular Signature Database C2 collection (MSigDB) (www.broadinstitute.org/gsea/msigdb/) were updated to official symbols using the illumina annotation file HumanHT-12_V3_0_R2_11283641_A, while the CR signatures were created according to the Table 2 of 3 and M.musculus to H.sapiens conversion obtained using the NCBI HomoloGene tool (http://www.ncbi.nlm.nih.gov/homologene).

Representation of differentially expressed genes in their Genomic localization and visual representation of their relationships with the different pathways were produced using CIRCOS software [28]. Statistical analysis of real time PCR and cell proliferation data was performed using Student's t test to compare the transcripts relative abundance in the CR group and WD group. P values <0.05 were considered statistically significant.
